# Occurrence of canine parvovirus in dogs from Henan province of China in 2009–2014

**DOI:** 10.1186/s12917-016-0753-1

**Published:** 2016-07-04

**Authors:** Zhanqin Zhao, Huisheng Liu, Ke Ding, Chunping Peng, Qiao Xue, Zuhua Yu, Yun Xue

**Affiliations:** Lab of Veterinary Microbiology, College of Animal Science and Technology, Henan University of Science and Technology, Luoyang, China; Lab of Medical Engineering, College of Medical Technology and Engineering, Henan University of Science and Technology, Luoyang, China

**Keywords:** Canine parvovirus, Canine distemper virus, Canine coronavirus, Parasites, Epidemiology

## Abstract

**Background:**

There is no information concerning the genotype of Canine parvovirus (CPV) currently circulating in Henan province, China. Therefore, the aim of the present study was to provide insights into the epidemiology and molecular characterization of CPV circulating in Henan province from 2009 to 2014.

**Results:**

Nineteen thousand nine hundred seven dogs from pet hospitals in the cities of Luoyang, Anyang, Jiaozuo, Sanmenxia, Xinxiang, Zhengzhou in Henan province between 2009 and 2014 were investigated. Over the 6-year period, 1169 CPV-positive cases were identified and the morbidity of CPV infection ranged from 4.16 to 8.06 %, although morbidity was not significant (*P* > 0.05) between 2009 and 2014. Factors associated with morbidity included sampling season, dog age, breed, vaccination status, and sex. CPV co-infection with coccidium (10.00 %), canine distemper virus (4.79 %), hookworm (2.40 %), canine coronavirus (1.11 %), roundworm (1.03 %), tapeworm (0.17 %) and *Babesia* spp. (0.09 %) were observed. The new CPV-2a variant was more prevalent than the new CPV-2b variant in Henan province. CPV 2c was not observed in this study.

**Conclusions:**

The epidemiology of CPV infection and identification of the circulating genotypes in Henan province, China from 2009 to 2014 determined that the new CPV-2a variant was more prevalent.

## Background

Canine parvovirus 2 (CPV-2), a member of the genus *Protoparvovirus* within the family *Parvoviridae* (the 2014 ICTV taxonomy, website: http://ictvonline.org/virusTaxonomy.asp), is a small, highly contagious, non-enveloped icosahedral virus that causes acute gastroenteritis mainly in domestic dogs [1–5]. CPV infection is responsible for large numbers of animal deaths worldwide and one of the most dangerous infectious diseases in young puppies aged 2–6 months. The infected dogs develops acute gastroenteritis, which is characterized by vomiting, fever, diarrhoea (from mucoid to haemorrhagic), haemorrhagic enteritis, myocarditis and leucopoenia [6, 7].

CPV contains a single-stranded DNA genome of approximately 5 kb, which encodes the capsid proteins 1, 2 and 3 (VP1, VP2 and VP3, respectively). VP1 and VP2 are splice variants and mostly identical in sequence, exclusive of a 143-amino-acid (aa) N-terminal region unique to VP1, Whereas VP3 is derived from the cleavage of VP2 at the 5’-terminal region by host proteases [8]. The full-length VP2 sequence (1755 bp) encodes the major capsid protein, which is 584 aa in length and plays an important role in determining viral host ranges, tissue tropisms, and genetic and antigenic properties [9, 10]. VP2 protein had been investigated as a candidate potential vaccine antigen due to its good immunogenicity [11]. CPV-2, first identified and described in 1978 in both the United States and Australia, is closely related to feline panleukopenia virus (FPV) [12]. A few years after its emergence, the original virus type CPV-2 was replaced worldwide by three new antigenic variants (i.e., CPV-2a, CPV-2b and CPV-2c) based on aa substitutions (CPV-2a: Val-555-Ile, Asp-305-Tyr, Ala-300-Gly, Ile-101-Thr and Met-87-Leu; CPV-2b: Ile-555-Val and Asp-426-Asn; CPV-2c: Asp-426-Glu) in the VP2 gene [12–18].

In China, CPV infections were first described in 1982 [9]. A few years later, widespread outbreaks of canine haemorrhagic enteritis occurred throughout the country and CPV infection emerged as an important zoonosis of dogs because of the high morbidity and lethality associated with the virus [9]. Recently, different CPV genotypes were observed in Heilongjiang, Jilin, Liaoning, Shandong, Hebei, Sichuan, Shenzhen, Gansu, Beijing, and Nanjing, as well as other areas in China [19–22]. However, no previous reports included information about the prevalent genotype of CPV in Henan province.

Vaccination is considered the most effective method to control CPV infection. However, antigenic differences may decrease the effectiveness of the vaccine based on the original antigenic type [23]. Therefore, it is important to elucidate the epidemiology and molecular characterization of CPV strains currently circulating in Henan province. Hence, in this study, we investigated the epidemiology of CPV infection among domestic dogs in Henan province and the molecular characterization of CPV by polymerase chain reaction (PCR) followed by direct sequencing of isolates collected from 2009 to 2014. The findings of this study constructed a basis for the further understanding of the evolution of CPV, will help to improve measures to prevent and control the spread of CPV and to developt effective vaccines against CPV infection.

## Methods

### Ethics statement

All animal procedures carried out in this study were approved by the Animal Care and Use Committee of Henan University of Science and Technology. Permission to collect samples was acquired from the owner of each animal. Samples were collected by a single skilled veterinarian. No specific permissions were required for collection of field samples because they were collected from public or non-protected areas.

### Samples collection

Nineteen thousand nine hundred seven dogs from pet hospitals in the cities of Luoyang, Anyang, Jiaozuo, Sanmenxia, Xinxiang, and Zhengzhou in Henan province from March 2009 to December 2014 were investigated. Among the 19,907 dogs, infections of dogs with active vomiting, fever, diarrhoea and haemorrhagic enteritis were detected using a CPV colloidal gold test strip (Bionote, Inc., Hwaseong, Gyeonggi Province, south Korea), according to the manufacturer’s protocol. Suspected CPV-positive dogs were confirmed by positive results of the CPV colloidal gold test strip. Then, faecal samples from suspected CPV-positive dogs were transferred to our laboratory for detection of canine distemper virus (CDV) and canine coronavirus (CCoV) using a CDV or CCoV colloidal gold test strip (Bionote, Inc.), respectively, and for detection of coccidium, ancylostome, roundworm, tapeworm, babesiosis and nematode by microscopy [24] according to the characteristic of these parasites. Additionally, the supernatant of faecal samples emulsified in 0.1 M phosphate-buffered saline (pH 7.2) and centrifuged (10,000 g for 10 min at 4–8 °C) [8] was collected for detection of the partial VP2 gene and complete VP2 gene by PCR.

### Detection of CPV genome

Viral DNA was prepared by boiling the supernatant for 10 min and chilling immediately on ice as previously described [25]. Specific primers (Table 1) for detection of the CPV genome were designed using Primer Premier 5.0 software (Premier Biosoft International, Palto Alto, CA, 85 USA) based on the conserved sequence of a previously published CPV genome sequence (GenBank accession no.: NC001539.1). The PCR reaction volume was 25 μl, which included 12.5 μl of PCR Master Mix (Sangon Biotech Company, Shanghai, China), 1 μl of each primer, 1 μl of supernatant, and 9.5 μl of ultrapure water. The PCR reaction included an initial denaturation step at 95 °C for 10 min followed by 30 cycles of 98 °C for 30 s, 55 °C for 30 s, and 72 °C for 40 s, and 72 °C for 10 min. A commercial vaccine was used as a positive control and sterile water was used as a negative control. The PCR-positive products were sent to Sangon Biotech Company for bidirectional single pass sequence analysis. The specificity of the sequences was then compared with those in the GenBank database (http://www.ncbi.nlm.nih.gov/genbank/) using the Basic Local Alignment Search Tool (www.ncbi.nlm.nih.gov/BLAST). Then, all PCR-positive sequences were aligned and three phylogenetic trees based on partial VP2 sequences were constructed by the Clustal W method using the MegAlign program (DNASTAR, Inc., Madison, WI, USA) [20].

**Table 1:** Primers for detection of the CPV genome and complete VP2 gene

Name	Sequence (5’ → 3’)	Position (VP2)	Product (bp)
CPVF	GAATCTGCTACTCAGCCACCAAC	463–485	560
CPVR	GTGCACTATAACCAACCTCAGC	1000–1021	
VP2F	AGAGACAATCTTGCACCAAT	2768–2787	1775
VP2R	ATGTTAATATAATTTTCTAGGTGCT	4519–4543	

### Detection of the complete VP2 gene

Viral DNA from diverse branches was prepared as described in this study. The primers (Table 1) for detection of the complete VP2 gene were designed using Primer Premier 5.0 software based on the consensus sequence of previously published CPV genomes (GenBank accession no.: JQ268284.1). The PCR reaction volume was 25 μl, which included 12.5 μl of PCR Master Mix (Sangon Biotech Company), 1 μl of each primer, 1 μl of supernatant, and 9.5 μl of ultrapure water. The PCR reaction included an initial denaturation step at 95 °C for 10 min followed by 30 cycles of 98 °C for 30 s, 48 °C for 50 s, and 72 °C for 120 s, and a final extension of 72 °C for 10 min. Sterile water was used as a negative control. The PCR positive products were sent to Sangon Biotech Company for bidirectional single pass sequence analysis. The specificity of the sequences was then compared with those in the GenBank database using BLAST algorithm.

### Sequence and phylogenetic analyses

The complete VP2 nucleotide sequences and aa sequences of our samples were aligned with reference CPV sequences from different animals and areas using MegAlign sequence alignment software (DNASTAR 6.0) [26]. The phylogenetic analyses based on complete VP2 nucleotide sequences from the CPV isolates in this study and 25 reference CPV strains were conducted by the neighbour-joining method and a Kimura 2-parameter model using Molecular Evolutionary Genetics Analysis (MEGA 5.0) software [27]. The confidence level of branching in the phylogenetic tree was evaluated by the bootstrap test based on 1000 resamplings. A bootstrap value of ≥ 70 % was considered significant for phylogenetic groupings [20]. To determine the genotype of the CPV isolates in this study, the complete VP2 aa sequences were aligned with reference CPV sequences (NCBI GenBank accession no.: M38245.1, M24003.1, M74849.1, KT156832.1, KT156834.1 and KT156833.1) using the DNAMAN software package (Lynnon Biosoft, San Ramon, CA, USA) [18]. The complete VP2 DNA sequences were also submitted to the GenBank database for assignment of accession numbers.

### Questionnaire survey

A questionnaire survey was created to further elucidate the epidemiology of CPV infection among domestic dogs between 2009 and 2014, as previously described with some modifications [28]. The questionnaire inquired about the followed topics: ‘CPV-positive’ (yes/no), ‘year’ (2009–2014), ‘month’ (spring, 3–5, summer, 6–8, autumn, 9–11, winter, 12–2), ‘age’ (1, 2, 3, 4, 5, 6, 6–9, 9–12, 12–36, >36 months), ‘sex’ (male or female), ‘purebred’ (yes/no), ‘vaccination’ (unvaccinated, intermittent vaccination: inoculation as part of an unofficial immune program, complete vaccination: inoculation as part of an official immune program), ‘co-infection’ (pathogen).

### Statistical analysis

Statistical analysis to assess significant differences of morbidity was performed using SPSS version 17.0 software. A *p*-value <0.05 was considered statistically significant.

## Results

### Morbidity of CPV infection in dogs from Henan province

**Fig. 1:**
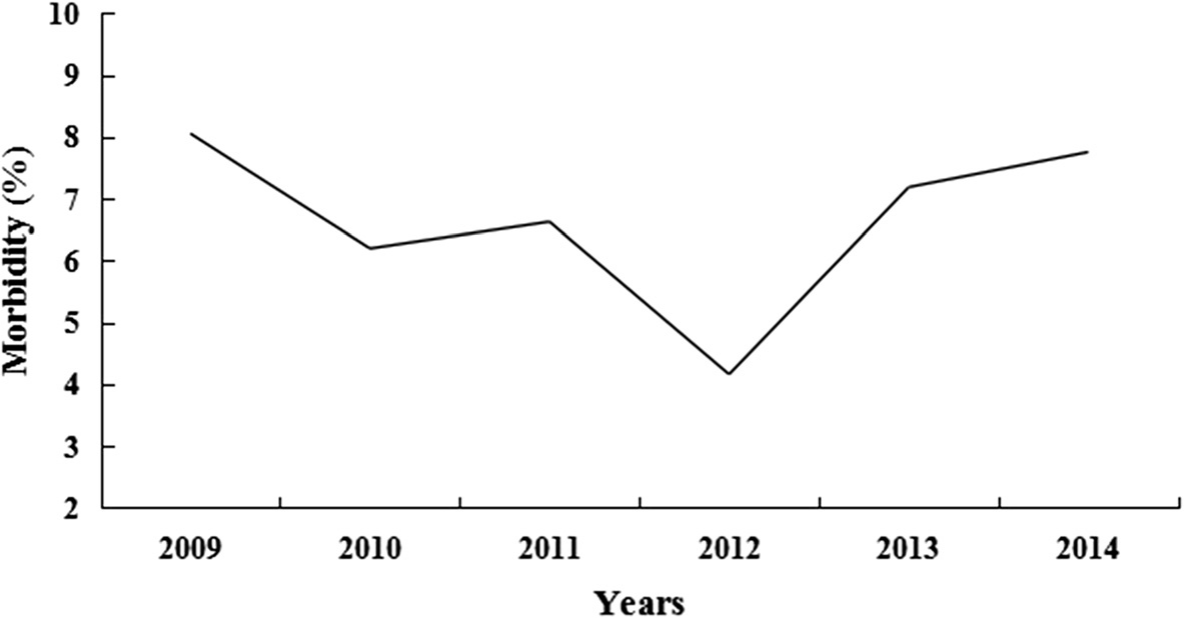
Annual distribution of morbidity of CPV infection. The morbidity rates were 51/633, 142/2291, 215/3242, 322/7746, 333/4629 and 106/1366 for 2009–2014, respectively. There was no significant difference in morbidity rates between 2009 and 2014 (*P* > 0.05, χ^2^ test)

**Fig. 2:**
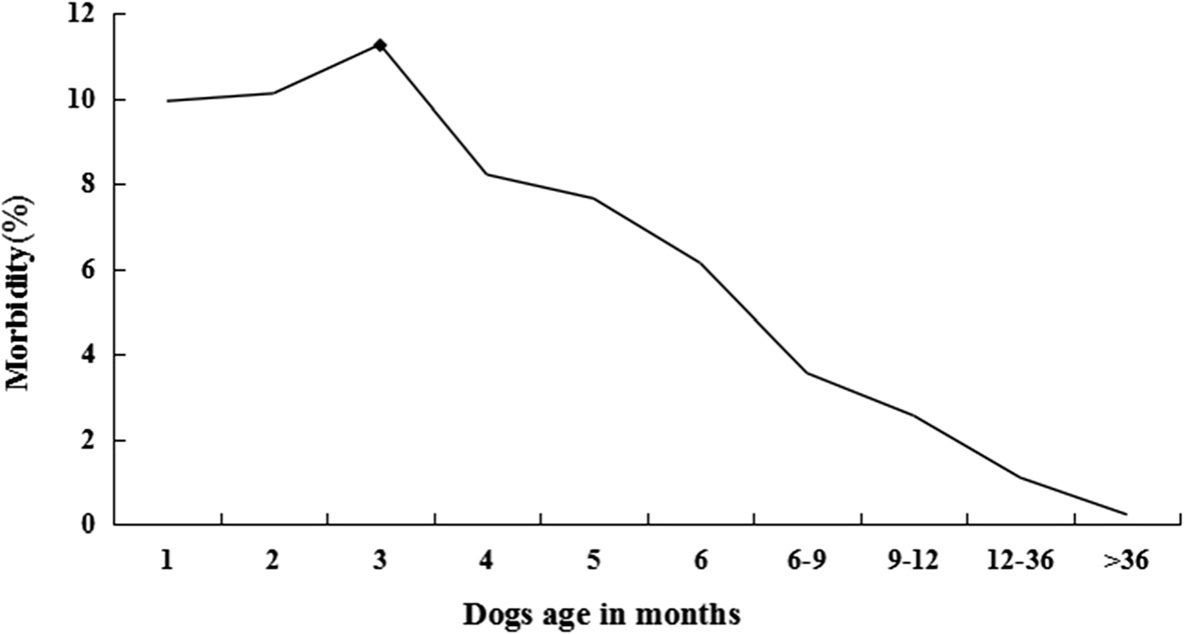
Distribution of morbidity of CPV infection in relation to dog age. The incidence of morbidity at different ages was 83/836, 319/3154, 291/2584, 145/1765, 116/1516, 75/1224, 68/1916, 41/1610, 24/2210 and 7/3092, respectively. The morbidity was relatively high in the first 4 months with the highest recorded in month 3 (◆). After month 3, morbidity gradually decreased. However, as a whole, *P*-value was <0.05 for morbidity in relation to dog age. Statistical analysis was performed using the χ^2^ test

**Fig. 3:**
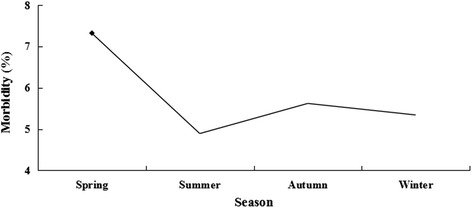
Distribution of morbidity of CPV infection in relation to the season. The incidence of morbidity was 435/5946, 260/5322, 251/4465 and 223/4174 for spring, summer, autumn and winter, respectively. As a whole, the morbidity was not significant (*P* > 0.05). The highest morbidity (◆) was recorded in the spring. Statistical analysis was performed using the χ^2^ test

One thousand one hundred seventy eight CPV-positive cases were identified by the CPV colloidal gold test strip. PCR analysis confirmed that 1169 (99.24 %) of these cases were CPV-positive. Of the 1169 partial VP2 sequences, 33 were submitted to the NCBI Genbank database under the accession numbers KF772192–KF772224. The morbidity of CPV infection between 2009 and 2014 was 5.87 % (1169/19907). The annual morbidity rate ranged from 4.16 to 8.06 % with the highest morbidity recorded in 2009 and the lowest in 2012 (Fig. 1), but morbidity was not significant (*P* > 0.05) between March 2009 and December 2014. In addition, we observed a significant influence of age on morbidity (*P* < 0.05, Fig. 2). Morbidity was relatively high among puppies during the first 4 month of life with the highest rate occurring in the third month. Afterward, the morbidity rate gradually decreased. Seasonal morbidity ranged from 4.89 to 7.32 % with the highest during the Spring (Fig. 3). However, as a whole, differences in morbidity according to season was not significant (*P* > 0.05). There was no significant difference in morbidity rates between male and female dogs (643/10937, 5.88 vs. 526/8970, 5.86 %, respectively, *P* > 0.05). The CPV-positive rate was significantly greater among purebred dogs compared to other dogs (1031/1169, 88.20 vs. 138/1169, 11.80 %, respectively, *P* < 0.05), and was highest among unvaccinated dogs (680/1169, 58.17 %), although not significantly (*P* = 0.069) as compared to intermittently vaccinated dogs (463/1169, 39.61 %), but significantly (*P* < 0.05) as compared to completely vaccinated dogs (26/1169, 2.22 %).

### Co-infection

Over the 6-years period of this study, 230 (19.67 %) of 1169 CPV-positive cases were co-infected with other pathogens, such as CDV, CCoV, coccidium (*Isospora*), hookworm (*Ancylostoma*), roundworm (*Toxocara*), tapeworm (*Dipylidium*) and *Babesia spp*. at rates of 4.79 % (56/1169), 1.11 % (13/1169), 10.00 % (117/1169), 2.40 % (28/1169), 1.03 % (12/1169), 0.17 % (2/1169) and 0.09 % (1/1169), respectively. Additionally, of the 1169 CPV-positive cases, 10 were co-infected with coccidium and hookworm, five with CDV and coccidium, and four with CCoV and coccidium.

### Sequence analysis

**Fig. 4:**
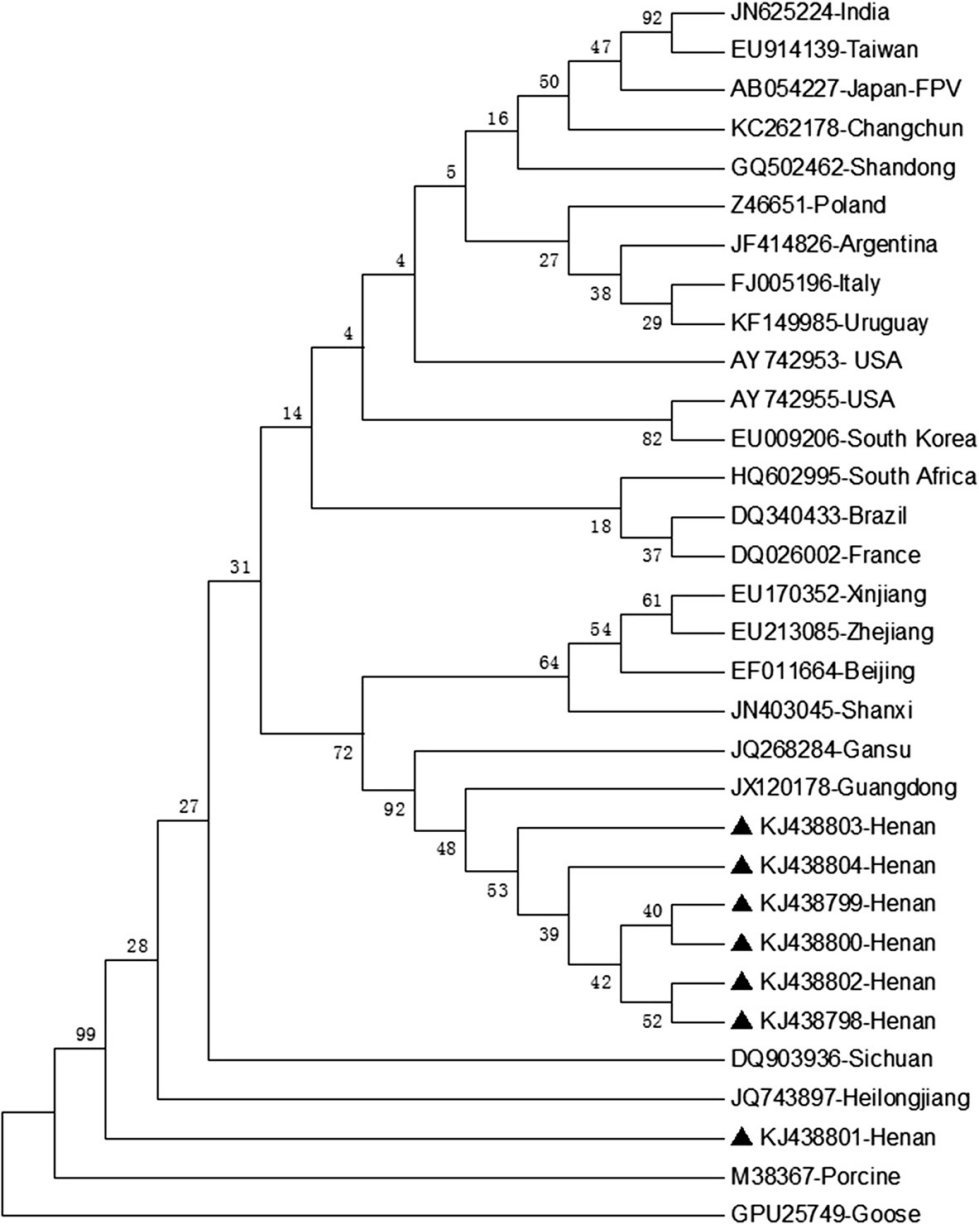
A Phylogenetic tree based on VP2 gene sequences of seven CPV isolates and 25 reference CPV strains by neighbor-joining method. ▲ indicates the seven CPV isolates from Henan province, China

Phylogenetic trees based on 1169 partial VP2 sequences amplified using CPVF/CPVR primers were constructed, which contained seven branches. One sample was selected from each branch for amplification of the complete VP2 gene, which suggested similarity of VP2 sequences of 99 % compared to those retrieved from the GenBank database. The seven complete VP2 nucleotide sequences (NCBI GenBank accession no.: KJ438798–KJ438804) and aa sequences were analysed using DNASTAR software, which revealed 99.4–99.9 % homology of nucleotide sequences and 99.1–99.8 % homology of aa sequences among the seven isolates; 98.4–99.9 % homology of nucleotide sequences and 97.6–100.0 % homology of aa sequences with CPV isolates from other provinces in China; 98.8–99.8 % homology of nucleotide sequences and 98.1–100.0 % homology of aa sequences with CPV isolates from other countries; 98.0–98.3 % homology of nucleotide sequences and 96.8–98.0 % homology of aa sequences with the FPV isolates; <60 % homology of nucleotide and aa sequences with porcine parvovirus; and <25 % homology of nucleotide and aa sequences with goose parvovirus. Phylogenetic tree analysis based on the seven complete VP2 nucleotide sequences (Fig. 4) revealed that the seven isolates were clustered to two branches. KJ438798-Henan, KJ438799-Henan, KJ438800-Henan, KJ438802-Henan, KJ438803-Henan and KJ438804-Henan were most closely related to the isolate (JX120178) from Guangdong province, southeast China, followed by the isolate (JQ268284) from Gansu province, northwest China; KJ438801-Henan was most closely related to the isolate (JQ743897) from Heilongjiang province, northernmost China, followed by the isolate (DQ903936) from Sichuan province, southwest China.

### Genotyping of CPV

Comparisons of the aa sequences among the seven isolates and the six reference strains revealed that Glu426, which is unique to strain CPV 2c, was not observed in any strain in this study. Mutations at aa 426 (N-426-D) and aa 297 (S-297-A) were found in one isolate (GenBank accession no.: KJ438800), which was typed as a new CPV-2b variant. The other isolates were typed as new CPV-2a variants due to the aa substitutions of N426 and A297 (Table 2).

**Table 2:** Deduced amino acid variations in VP2 in the seven CPV isolates from Henan province, China

Accession no	Origin	Place of amino acid sites									Gene types
		267	297	322	324	334	341	348	426	440	
Reference											
M38245	U.S.	F	S	T	Y	A	P	S	N	T	cpv-2
M24003	U.S.	F	S	T	Y	A	P	S	N	T	cpv-2a
M74849	U.S.	F	S	T	Y	A	P	S	D	T	cpv-2b
KT156832.1	China	Y	A	T	I	A	P	S	E	T	cpv-2c
KT156834.1	China	Y	A	T	I	A	P	S	N	A	new cpv-2a
KT156833.1	China	Y	A	T	I	A	P	S	D	A	new cpv-2b
This study											
KJ438798	Henan	Y	A	-	I	G	S	-	N	A	new cpv-2a
KJ438799	Henan	Y	A	S	I	-	S	-	N	A	new cpv-2a
KJ438800	Henan	Y	A	-	I	-	-	-	D	A	new cpv-2b
KJ438801	Henan	F	A	S	I	G	-	C	N	T	new cpv-2a
KJ438802	Henan	Y	A	-	I	-	S	-	N	A	new cpv-2a
KJ438803	Henan	Y	A	-	I	-	S	-	N	A	new cpv-2a
KJ438804	Henan	Y	A	-	I	-	-	-	N	A	new cpv-2a

Identical amino acids are represented by -

## Discussion

This study was the first attempt to evaluate the occurrence of CPV infection among domestic dogs between 2009 and 2014 in Henan province, China. A diagnosis of CPV infection based on clinical signs and the CPV colloidal gold test strip is rapid and often used in pet hospitals in China. Of 1178 faecal samples, 1169 (99.24 %) were CPV-positive by PCR assay, suggesting a good correlation between the antigen-detection test and molecular methods, with only nine samples being recognized as false positives. In this study, the morbidity of CPV infection was 5.87 % (1169/19907), which was lower than in recent reports from China [20, 29]. The sample size in this study was much larger than samples size described previously, thus these results should represent a more accurate estimate of morbidity of CPV infection. Additionally, the χ^2^ test results showed that the morbidity of CPV infection fluctuated only slightly (*P* > 0.05) within the 6-years period of this study (2009–2014).

The results of this study showed that morbidity in the spring (months 3–5) and autumn (months 9–11) were relatively higher than observed during the other seasons, but the morbidity in Heilongjiang province differed from that reported for other provinces [29], suggesting variations in morbidity according to the season in different regions of China. In addition, our work demonstrated a clear association of CPV infection with dog age (*p* < 0.05), which in accordance with the results reported by Geng et al. [29] and Cavalli et al. [30]. Although the causes of this tendency have not yet been fully elucidated, we hypothesise that the low morbidity in dogs aged < 30 days most likely resulted from acquired maternal antibody, while the high morbidity in dogs aged 30–120 days most likely resulted from decreasing maternal antibody concentrations and stress due to weaning, and the low morbidity in dogs aged >120 days was most likely attributable to the development of adaptive immune responses [31, 32]. The finding of more male dogs in this study was consistent with those in other parts of the world, suggesting that there also is a preference of male over female dogs in Henan province [28]. Besides, to the best of our knowledge, this study is the first to report that morbidity (5.88 %) in domestic male dogs was similar to that (5.86 %) in domestic female dogs. CPV infection occurs in both unvaccinated and vaccinated dogs, in accordance with the finding of recent reports in China [29, 33]. In this study, the CPV-positive rate was highest among unvaccinated dogs, followed by intermittently vaccinated dogs and completely vaccinated dogs, suggesting that vaccines used in Henan province may play an important role in prevention and control CPV infection. These results were different from those of previous reports from other areas of China. Our work also indicated that the CPV-positive rate was higher among purebred dogs as compared to mutts. This result suggested that mutts may have greater resistance to CPV, which should be investigated in future studies.

In this study, CPV infection was identified alongside other pathogens in 230 (19.67 %) of 1169 CPV-positive samples. The pathogens that accompanied CPV were coccidium, CDV, CCoV, hookworm, roundworm, tapeworm and *Babesia spp.*, suggesting that these pathogens should be considered in vaccination programs to control CPV outbreaks [34]. The co-infection of CPV with CDV or CCoV in this study was in agreement with previous study in China [29] and other countries [30, 35, 36]. Additionally, studies in China [37] and France [38] suggested that dogs harbouring roundworm or trichomonads were especially susceptible to CPV infection. Also, this study is the first to report the co-infection of CPV with coccidium, hookworm, tapeworm or *Babesia spp.*, and co-infection of two pathogens with CPV was also found in one sample.

Point mutations in the VP2 protein have been associated with the CPV types because the fragment encodes for at least one informative aa (residue 426) of the VP2 protein, which in used to differentiate CPV2a (Asn), CPV2b (Asp) and CPV2c (Glu) [9]. The Ser297Ala mutation was used as a marker of the new CPV-2a/2b variant [29]. CPV-2c was first identified in Jilin province, and subsequently identified in Shandong province, Heilongjiang province and Beijing [19, 29, 33]. CPV-2c was not detected in this study, while the new CPV-2a variant is more prevalent than new CPV-2b variant in Henan province. In this study, the prevalence of new CPV-2a and new CPV-2b is in agreement with most of the reports in other provinces of China [6, 8, 9, 20, 29, 33, 39, 40] and other countries [26, 41–45]. The presence of the new CPV-2a and CPV-2b variants in this study suggests that CPV-2 strains circulating in Henan province also exhibit genetic variations.

VP2 encodes a viral capsid protein that is the major structural protein of CPV-2 and is involved in the host immune response. Therefore, a small number of mutations may result in increased pathogenicity [26]. In this study, the aa sequences from the seven VP2 gene products revealed little variability at residues 322, 334 and 348, which was basically consistent with reference CPV sequences. The aa sequences from seven VP2 gene products revealed great variability at residues 267, 324, 341 and 440. Residue 267, located between loops 1 and 2 of the VP2 protein, is the main component of the antigen. However, a mutation to residue 267 may not influence the antigenicity of CPV because it is not exposed on the capsid surface, as was reported for strain CPV-2a from China and Thailand and strain CPV-2b from Vietnam [45]. Residue 324 is subject to positive selection and may influence the aa sequences at residue 323, which is known to be involved in host range via binding with the canine transferrin receptor. A mutation at residue 323 may influence interactions between residues in neighbouring loops of either the same VP2 molecule or the threefold-related VP2, thereby greatly decreasing replication in canine cells [26]. The Y-324-I mutation in CPV-2a strains has been reported in China [8, 20, 39] and other countries [43, 45]. Interestingly, variations of residue 440 (within the GH loop) occurred. Reportedly, the high substitution rate in this region is associated with the evolution of antigenic variants in circulating parvovirus types [46], such as strains CPV-2a and CPV-2b from other provinces in China [9, 29]. However, the significance of a mutation at residue 341 is unclear and warrants further investigations.

Previous reports suggested that the new CPV-2a variant was the most prevalent in China, followed by CPV-2b and CPV-2c, in accordance with the results of the present study. However, it is necessary to collect more samples form Henan province for further genotyping to improve our understanding of the evolution of CPV in China.

## Conclusions

Our results disclosed novel epidemiological information of CPV infection in Henan province. VP2 sequences data revealed that the new CPV-2a variant is more prevalent than the new CPV-2b variant in Henan province. CPV-2c was not observed in this study. The findings of this study are expected to facilitate a better understanding of the current status of CPV-2 infection among dogs in China.

## Abbreviations

CCoV, canine coronavirus; CDV, canine distemper virus; CPV, canine parvovirus; FPV, feline panleukopenia virus; PCR, polymerase chain reaction
